# Effect of Amino Trimethylene Phosphonic Acid and Tartaric Acid on Compressive Strength and Water Resistance of Magnesium Oxysulfate Cement

**DOI:** 10.3390/ma18153473

**Published:** 2025-07-24

**Authors:** Yutong Zhou, Zheng Zhou, Lvchao Qiu, Kuangda Lu, Dongmei Xu, Shiyuan Zhang, Shixuan Zhang, Shouwei Jian, Hongbo Tan

**Affiliations:** 1 State Grid Zhejiang Electric Power Co., Ltd., Research Institute, Hangzhou 310014, China; zhouzheng1020@zju.edu.cn (Z.Z.); 3110103124@zju.edu.cn (L.Q.); 15703919191@163.com (K.L.); 331472@whut.edu.cn (D.X.); 345088@whut.edu.cn (S.Z.); 2 State Key Laboratory of Silicate Materials for Architectures, Wuhan University of Technology, Wuhan 430070, China; zsxwhut@163.com (S.Z.); jianshouweiwhut@163.com (S.J.); thbwhut@whut.edu.cn (H.T.)

**Keywords:** chelation, compressive strength, water resistance

## Abstract

Organic acids could act as retarders in magnesium oxysulfide (MOS) systems, not only delaying setting and improving fluidity but also enhancing compressive strength and water resistance. These effects are generally attributed to both the presence of H^+^ ions and anion chelation. However, the enhancement efficiency of different organic acids in MOS systems varies significantly due to differences in their molecular structures. To determine the underlying mechanism, this study comparatively investigated the effects of amino trimethylene phosphonic acid (ATMP) and tartaric acid (TA) on the setting time, fluidity, compressive strength, and water resistance of the MOS system, with the two additives incorporated at mole ratios to MgO ranging from 0.002 to 0.006. The mechanism behind it was revealed by discussion on the hydration heat, hydrates, and pH value. Results showed that both ATMP and TA could effectively improve the fluidity, delay the setting process, and enhance the mechanical properties, including strength and water resistance. At a mole ratio of 0.006, the incorporation of ATMP increased the 28 d compressive strength and the softening coefficient by 214.12% and 37.29%, respectively, compared with the blank group. In contrast, under the same dosage, TA led to an increase of 55.13% in the 28 d strength and 22.03% in the softening coefficient. Furthermore, hydration heat, product analysis, and pH measurements indicated that both ATMP and TA inhibited hydration during the initial hours but promoted hydration at later stages. The potential reason could be divided into two aspects: (1) H^+^ ions from ATMP and TA suppressing the formation of Mg(OH)_2_; (2) anion chelation with Mg^2+^ in the liquid phase, leading to a supersaturated solution with higher saturation, which further hindered Mg(OH)_2_ formation and facilitated the later development of 5Mg(OH)_2_·MgSO_4_·7H_2_O (517 phase). By contrast, under the same mole dosage of H^+^ or anions, the enhancement in compressive strength as well as the water resistance is superior when using ATMP. This was owing to its stronger chelating ability of ATMP, which more effectively inhibited Mg(OH)_2_ formation and then promoted the formation of the 517 phase. These findings confirm that the chelating ability of anions exerts an important impact on the retarding effect as well as the enhancement of strength in MOS systems.

## 1. Introduction

Magnesium oxysulfide (MOS) cement, an ambient-curing cementitious material, is composed through the reaction of light-burned magnesium oxide and heptahydrate magnesium sulfate. Due to its low CO_2_ emissions during raw material calcination and the relatively low calcination temperature, MOS cement has been regarded as a sustainable binder with a low carbon footprint [1,2,3]. Numerous studies have shown that MOS cement-based materials exhibit superior properties, including lightweightness, non-combustibility, low thermal conductivity, and good fire resistance, making them highly promising for applications in lightweight thermal insulation and refractory materials [4,5,6]. However, its practical application was significantly limited owing to the inferior mechanical performance and insufficient water resistance [7,8,9].

In the literature, silica gel [10], silica fume [11], ferric sulfate [12], and magnesium slag [13] could improve both strength and water resistance, and this improvement was attributed to the generation of gels, such as magnesium silicate hydrate (M-S-H) gel, Fe(OH)_3_ gel, and calcium silicate hydrate (C-S-H) gel, which offered excellent water resistance and contributed to microstructural densification through their filling effect. Other studies confirmed that organic acids could also improve both the strength and water resistance of MOS, such as citric acid [14,15], phosphate-based acid [16,17], and tartaric acid [18]. One reason is that the formation of Mg(OH)_2_, which is detrimental to strength development and water resistance, was inhibited by the H^+^ ions provided from these acids [19]. By hindering the formation of Mg(OH)_2_, the conditions become more favorable for the generation of 5Mg(OH)_2_·MgSO_4_·7H_2_O (5·1·7), which is contributing positively to both strength development and water resistance. The other reason was that these anions in acid could chelate with Mg^2+^, thereby hindering the formation of Mg(OH)_2_, which is positive for the generation of the 5·1·7 phase. Furthermore, recent research has presented the difference between sodium citrate and citric acid in enhancing MOS cement [20]. The experimental results revealed that there was no obvious difference in the mechanical strength values; the proportion of Mg(OH)_2_ in hydrates in the citric acid sample was 7.64%, and that of sodium citrate was 10.26%, much higher than that of citric acid; this further confirmed the important role of H^+^ in hindering the formation of Mg(OH)_2_. Organic salts, such as calcium citrate and sodium citrate without H^+^, could also greatly improve the strength and water resistance, and this further proved the important role of the chelation with Mg^2+^, not only hindering the formation of Mg(OH)_2_ but also facilitating the formation of the 5·1·7 phase [21]. Based on research, hindering the formation of Mg(OH)_2_ by offering H^+^ and chelating with Mg^2+^ to finally promote the formation of the 5·1·7 phase has been accepted as a popular way to enhance the mechanical properties of MOS cement.

In recent years, organic phosphonic acid compounds have emerged in the modification of cement-based materials due to their excellent chelating properties with metal ions. Amino trimethylene phosphonic acid (ATMP), as a typical organic phosphonic acid with multiple phosphonic acid groups and amino groups in its molecular structure, could obviously improve the compressive strength and water resistance of MOS [22], and the mechanism could be revealed from two aspects of H^+^ reaction and Mg^2+^ chelation, which were mentioned above. To further understand the synergy between these two aspects, the effect of ATMP and tartaric acid with various mole dosages on the mechanical strength, water resistance, and hydration process of MOS cement was comparatively discussed, and the mechanisms behind it were revealed. The results were expected to give a deep insight into the mechanisms behind the improvement in water resistance and compressive strength by adding organic acid, as well as offer a useful reference for the selection and design of modifiers for magnesium oxychloride (MOS) cement.

## 2. Materials and Test Methods

### 2.1. Materials

**Figure 1:**
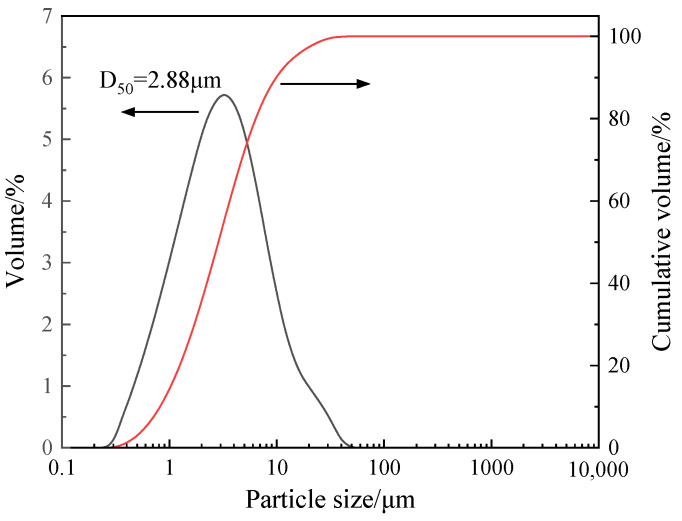
Particle size distribution curve of MgO.

**Figure 2:**
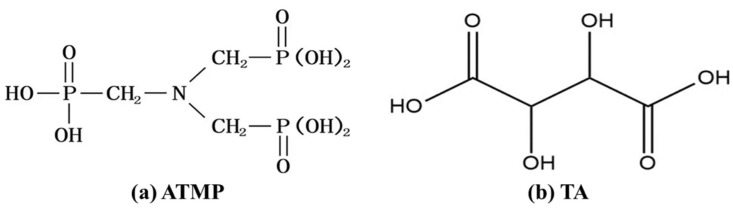
Molecular structural formula of ATMP and TA.

The materials mainly used, including light-burned magnesium oxide (MgO) and magnesium sulfate heptahydrate (MgSO_4_·7H_2_O), were produced by Sinopharm Chemical Reagent Co., Ltd. (Shanghai, China). The particle size distribution of MgO is presented in Figure 1. Moreover, amino trimethylene phosphonic acid (ATMP) and tartaric acid (TA) were used to modify MOS cement, and the molecular structures of ATMP and TA are shown in Figure 2. ATMP was purchased from Sandu Chemical Co., Ltd. (Hangzhou, China). TA was obtained from Aladdin Biochemical Technology Co., Ltd. (Shanghai, China).

### 2.2. Sample Preparation

**Figure 3:**
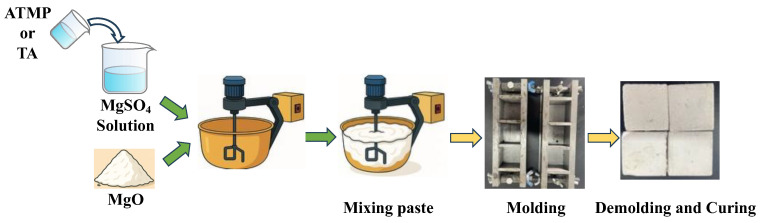
Sample preparation process of MOS.

The mix ratio of MOS cement is shown in Table 1, and the sample preparation process is shown in Figure 3. MgSO_4_·7H_2_O and water were mixed evenly to obtain a magnesium sulfate solution, and then ATMP and TA were dissolved in the magnesium sulfate solution; stirring was continued for 2 min to make them evenly dispersed. After that, MgO was mixed with the prepared solution to prepare a fresh MOS paste, which was then poured into a 40 mm × 40 mm × 40 mm mold, cured at room temperature for 24 h, and subsequently demolded. These fresh MOS pastes were also used for measurements of setting time and fluidity in accordance with Chinese standards GB/T 8077-2023 and GB/T 1346-2011 [23,24,25]. Placed the demolded samples in a curing room with a temperature of 20 °C and a humidity of 65% to continue curing for measurements. When the samples were cured to the specified age, measurements of the compressive strength and softening coefficient were carried out in compliance with Chinese standards GB/T 17671-2021 and GB/T 4111-2013 [26,27]. After that, small pieces from the compressive strength measurement were soaked in isopropanol for 3 days to terminate hydration, and then they were put in a vacuum oven to dry to a constant weight. Then, they were ready for SEM testing. The remaining samples were ground to pass through a 200-mesh sieve for XRD testing.

### 2.3. Test Methods

(1) Hydration heat

The fresh MOS paste was also used for hydration heat. About 3 g of fresh paste was put into a 10 mL glass bottle, and then the bottle was placed into the machine (TAM air 8, TA Instruments, New Castle, DE, USA), and the data was recorded automatically; all procedures were operated at 20 °C.

(2) Conductivity and pH value of MOS suspension

About 20 g of fresh paste and 200 g of deionized water were mixed to be even, and immediately after, pH value and conductivity were tested using a Mettler SD-20 pH meter and a Mettler S500-B Conductivity Tester (METTLER TOLEDO, Columbus, OH, USA) automatically every 5 min within the first 60 min, followed by every 30 min.

(3) Conductivity of solution

The measurement of conductivity was carried out with a Mettler S500-B Conductivity Tester, and the data was logged automatically via the instrument. Next, 1.0 mol/L MgSO_4_·7H_2_O solution, 1.0 mol/L MgO solution, 0.10 mol/L ATMP solution, and 0.10 mol/L TA solution were prepared, respectively, for the measurement of conductivity. A flow rate of 6.0 mL/min was maintained using peristaltic pumps during the addition process.

(4) Hydration analysis

An XRD test was conducted to detect the phase composition of the sample; the machine was manufactured by Bruker, Bremen, Germany (D8 Discover X-ray diffractometer) [28,29]. The scanning speed was 5°/min, and the test angle 2θ range was 5–60°. A QUANTA FEG 450 field emission environmental scanning electron microscope (FELMI-ZFE, Graz, Austria) was used to observe the microstructure of the sample.

## 3. Results and Discussion

### 3.1. Fluidity and Setting Time

**Figure 4:**
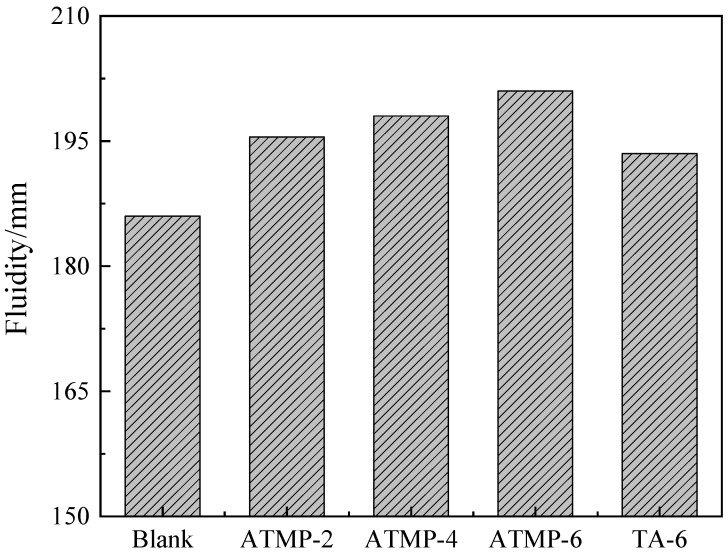
Fluidity of MOS paste with ATMP and TA.

Figure 4 presents the test results of the fluidity of MOS paste with ATMP and TA. According to the figure, it was found that both ATMP and TA were able to improve the fluidity of MOS paste. As the dosage of ATMP increased, the fluidity also increased. Compared to TA-6, all groups with ATMP showed higher fluidity. It was noted that the ATMP-2 group, with the same moles of H^+^ added as the TA-6 group, presented higher fluidity than that of TA-6, and this indicated that the anion ion played the main role in fluidity improvement. Comparing ATMP-6 with TA-6, it was seen that with the same mole dosage of anions, ATMP showed a higher capacity of fluidity improvement than TA.

**Figure 5:**
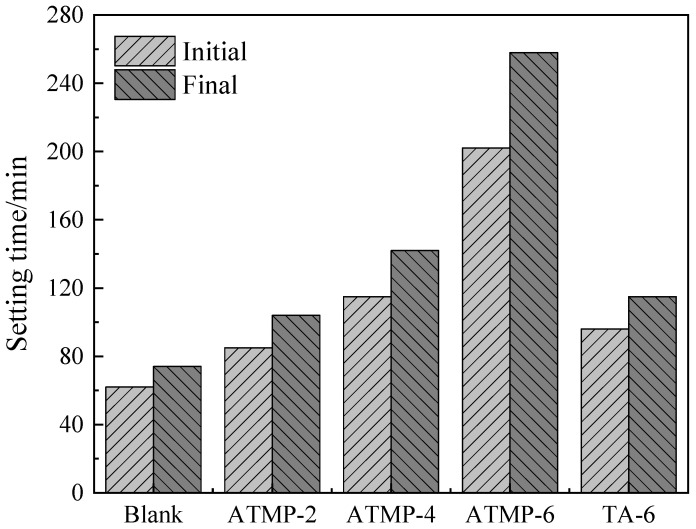
Setting time of MOS paste with ATMP and TA.

Figure 5 shows the influence of ATMP and TA on the setting time of MOS. From the figure, it can be seen that with the increase in dosage of ATMP, the setting time was increased, and TA could also prolong the setting time. These results confirmed that ATMP and TA acted as retarders in MOS, in agreement with the results in the literature [17]. Furthermore, the initial and final setting times of the blank group were 62 min and 74 min, respectively. In comparison, the initial setting time of the ATMP-2 group was extended by 37.09% to 85 min, and the same result could be observed in the final setting time, which was extended by 40.54% to 104 min. The initial setting time of the ATMP-6 group was 202 min and the final setting time was 258 min, which were extended by 225.81% and 248.65%, respectively, compared with the blank group. As for the TA-6 group, the initial and final setting times were 96 min and 115 min, respectively, which were extended by 54.84% and 55.41%, respectively, compared with the blank group. Moreover, with the same mole dosage of H^+^, it was found that TA-6 showed a slightly stronger ability to delay the setting of MOS than that of ATMP-2, about 11 min delay in both initial and final setting time. With the same mole dosage of anions, ATMP-6 presented a greatly stronger ability to delay the setting than that of TA-6, with an increase in initial setting time from 96 min in TA-6 to 202 min in ATMP-6, with an increase of 110%, and final setting time increased by 124%.

### 3.2. Compressive Strength

**Figure 6:**
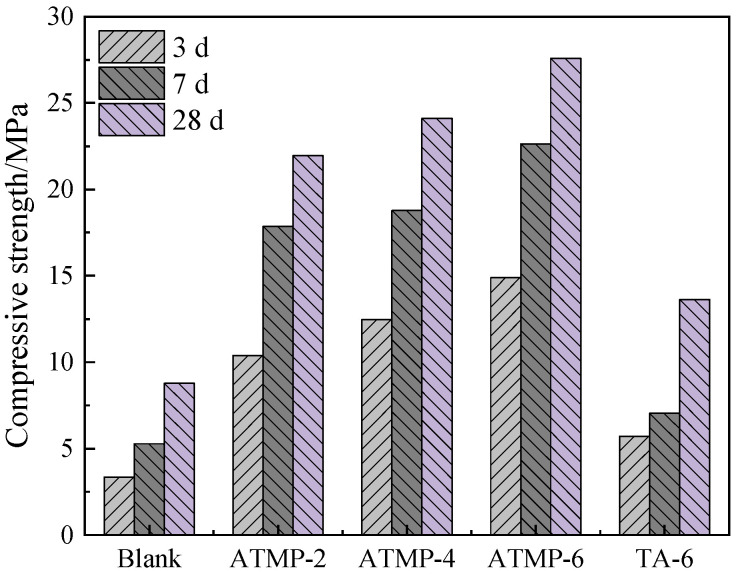
Effect of ATMP and TA on the compressive strength of MOS.

The compressive strength of MOS added with ATMP and TA was tested, and the results are shown in Figure 6. From the figure, with the increased dosage of ATMP, compressive strength at all curing ages was promoted. Compared to the blank, both ATMP and TA could increase the mechanical strength at 3 d, 7 d, and 28 d; to be specific, ATMP-2 increased 3 d strength from 3.35 MPa to 10.38 MPa, with an increase of 209.85%, and those of ATMP-4, ATMP-6, and TA-6 increased by 272.24%, 344.48%, and 70.45%, respectively. At the 28 d age, the strength of the blank group, ATMP-2, ATMP-4, ATMP-6, and TA-6 was 8.78 MPa, 21.94 MPa, 24.1 MPa, 27.58 MPa, and 13.62 MPa, respectively; compared to the blank group, there was an increase of 149.89% in ATMP-2, 174.48% in ATMP-4, 214.12% in ATMP-6, and 55.13% in TA-6. Furthermore, with the same mole dosage of H^+^, the enhancement of ATMP in compressive strength is more significant than TA at all curing ages; the same result was found in the comparison of ATMP-6 and TA-6, indicating that the anions played the main role in enhancing compressive strength.

### 3.3. Water Resistance

**Figure 7:**
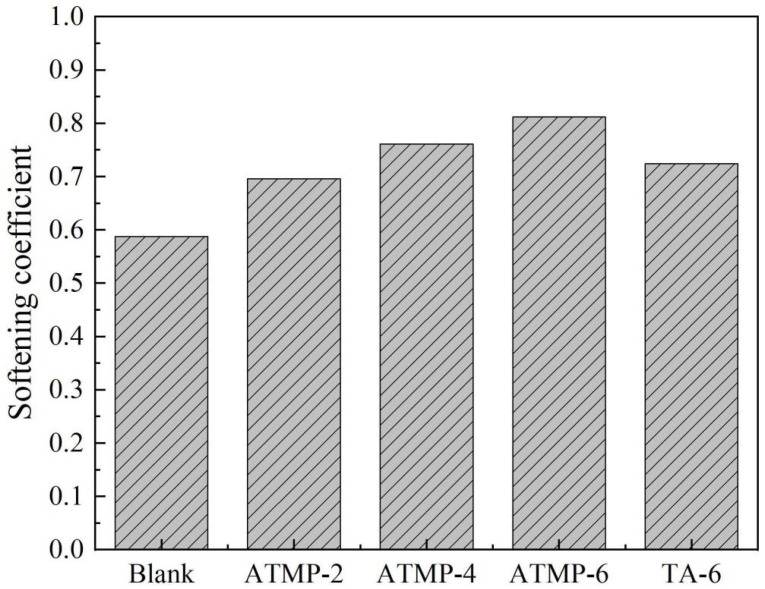
Effect of ATMP and TA on the water resistance of MOS.

The softening coefficients of MOS with ATMP and TA were tested, and the results are presented in Figure 7. It was obviously found that with the increase in ATMP dosage, the softening coefficient was greatly improved. Compared with the blank group, the softening coefficient of ATMP-2 increased from 0.59 to 0.70, with an increase of 18.64%. The softening coefficient of ATMP-6 was 0.81, which was 37.29% higher than that of the blank group. The softening coefficient of TA-6 was 0.72, which was 22.03% higher than that of the blank group. These results showed that both ATMP and TA could promote the water resistance of MOS paste. Furthermore, with the same mole dosage of H^+^, the softening coefficient of ATMP-2 was slightly higher than that of TA-6. With the same mole dosage of anions, ATMP-6 displayed a value 12.5% higher than TA-6. This result also indicates that anions mainly influence the water resistance of MOS [30,31].

### 3.4. Hydration Heat

**Figure 8:**
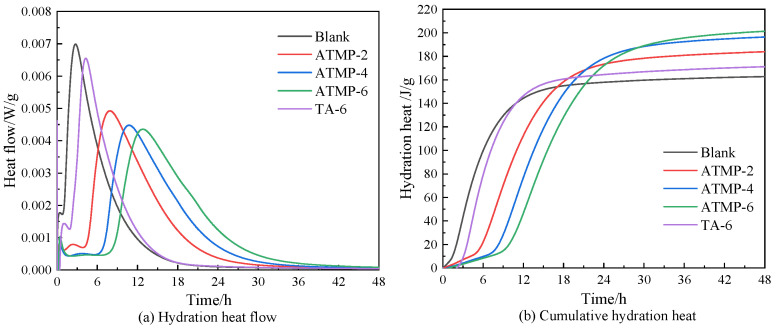
Hydration heat of MOS with ATMP and TA.

In order to evaluate the effect of ATMP and TA on the hydration process of MOS, hydration heats within 48 h were measured, and the results are presented in Figure 8. As can be seen in Figure 8a, it was found that both ATMP and TA could delay the heat release of MOS. In the blank group, the peak appeared at 2.75 h, and those of ATMP-2, ATMP-4, ATMP-6, and TA-6 were 7.93 h, 10.73 h, 12.87 h, and 4.22 h, respectively, and so were prolonged by 188.36%, 290.2%, 362.91%, and 53.45%, compared to the blank group. The highest value of heat flow in the blank group was 0.007 W/g, and those for ATMP-2, ATMP-4, ATMP-6, and TA-6 were 0.0048 W/g, 0.0045 W/g, 0.0044 W/g, and 0.0065 W/g, respectively, and so were reduced by 31.43%, 35.71%, 37.14%, and 7.14%. Furthermore, a clear dissolution equilibrium period was found in the MOS hydration process in the presence of ATMP, and with the increase in ATMP dosage, this period was gradually prolonged. However, the blank group and TA-6 group almost did not have a dissolution equilibrium period. Moreover, with the same mole dosage of H^+^, ATMP-2 was delayed by 3.71 h of peak appearance time compared to TA-6, indicating that ATMP-2 played a much stronger role in retarding MOS than TA-6. With the same mole dosage, ATMP-6 showed an 8.65 h delay in peak appearance time compared to TA-6. This indicates that the anions in ATMP show a much stronger ability to delay the hydration process of MOS.

Figure 8b shows the release of the total hydration heat in MOS added with ATMP and TA. It was found that the total hydration heat of samples with ATMP and TA was lower than that of the blank group at the beginning. As shown in Table 2, at 10.8 h, TA-6 showed the same cumulative hydration heat as the blank group; before 10.8 h, both TA and ATMP reduced the cumulative hydration heat. After 24 h, the cumulative hydration heat of these samples with ATMP and TA was higher than that of the blank group. This indicates that these chemicals hindered the hydration heat release at the beginning while accelerating the hydration release after 24 h, in agreement with results in the literature [17]. By contrast, at 4 h, the cumulative hydration heat of the ATMP-2 sample was 9.77 J/g, and it was much lower than that of TA-6; this indicated that with the same mole dosage of H^+^, ATMP-2 presented a stronger ability to hinder the hydration of MOS than that of TA-6 at the beginning. At the age of 4 h, the cumulative hydration heat of ATMP-6 was 4.92 J/g, and that of TA-6 was 33.24 J/g, which is much higher than that of ATMP-6; this indicates that with the same mole dosage, ATMP-6 shows a stronger ability than TA-6 to hinder the hydration of MOS at the beginning. Furthermore, at 24 h, ATMP-2 was 173.68 J/g, and TA-6 was 163.97 J/g, both of which are higher than the blank group; this indicates that with the same mole dosage of H^+^, ATMP-2 shows a stronger ability to accelerate hydration than TA-6 after 24 h. For ATMP-6, the 24 h hydration heat was 172.25 J/g, and it was higher than that of TA-6; this indicates that with the same mole dosage, ATMP-6 shows a stronger ability to accelerate hydration than TA-6 after 24 h.

Based on the discussion above, it was confirmed that the presence of ATMP and TA initially hindered the hydration process of MOS. However, at later stages, both additives were found to accelerate hydration. Compared to TA, ATMP exhibited a stronger ability to inhibit early-stage hydration and enhance later-stage hydration at the same mole concentration of H^+^. Furthermore, under equal mole dosages, ATMP showed a more pronounced inhibitory and accelerating effect than TA. These results suggest that the anion in ATMP has a more significant impact than that in TA in influencing the hydration of MOS.

### 3.5. Hydrates Analysis

**Figure 9:**
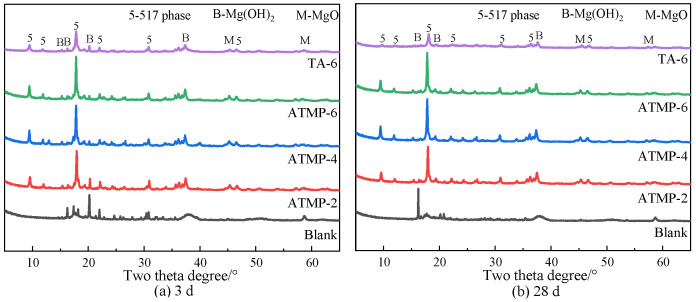
Effect of phase composition of MOS hydrated for 3 d and 28 d.

Hydrates of MOS hydrated at 3 d and 28 d were analyzed by XRD, and the results are shown in Figure 9. From the figure, the characteristic peaks of 5Mg(OH)_2_·MgSO_4_·7H_2_O (517 crystal phase), Mg(OH)_2_, and MgO were observed, indicating that the main hydrates of MOS are 5Mg(OH)_2_·MgSO_4_·7H_2_O and Mg(OH)_2_. From Figure 9a, the highest intensity of the Mg(OH)_2_ characteristic peak was observed in the blank group, and this indicated that ATMP and TA could hinder the formation of Mg(OH)_2_ and induce the formation of the 517 crystal phase. With the increase in dosage of ATMP, the intensity of the characteristic peak of the 517 crystal phase was increased. By contrast, at the same mole dosage of H^+^, the intensity of the 517 phase peak in ATMP-2 was higher than that observed in TA-6. Similarly, at the same mole dosage, the 517 phase peak intensity in ATMP-6 was significantly higher than that in TA-6. These results were consistent even at the 28 d mark, as presented in Figure 9b.

**Figure 10:**
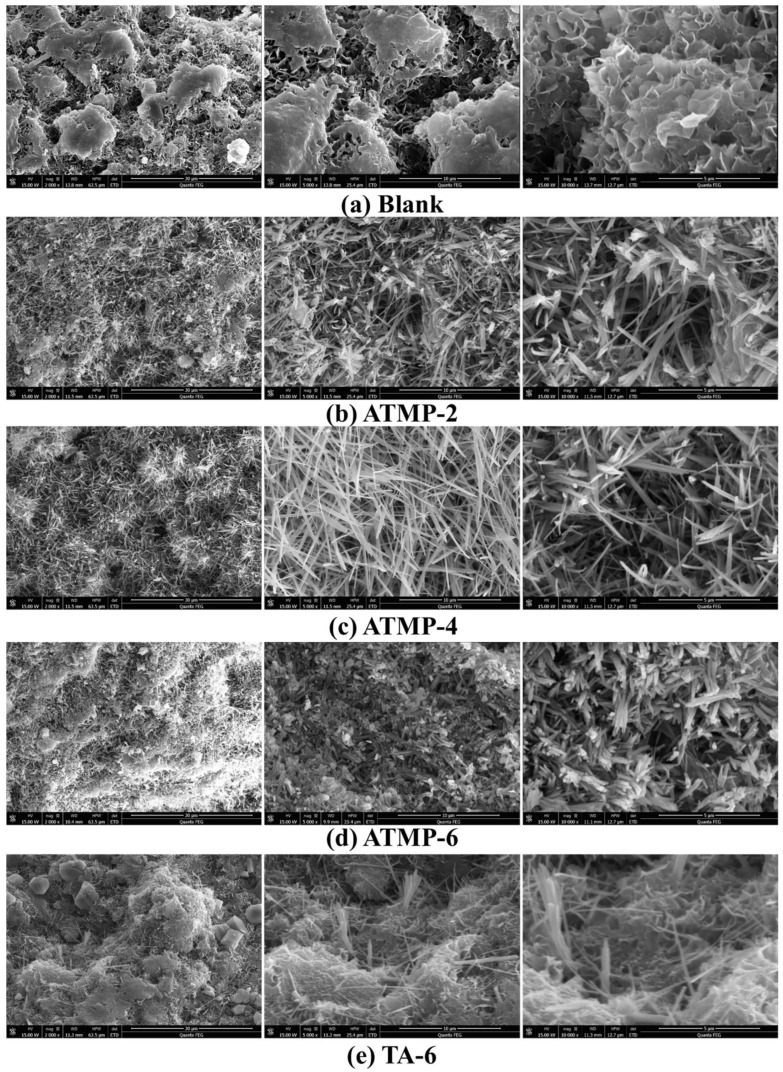
SEM images of microstructures of MOS hydrated for 28 d.

Figure 10 shows the SEM image of MOS hydrated with ATMP and TA for 28 d. From Figure 10a, a lot of petal-shaped Mg(OH)_2_ in the blank group was found, and its microstructure is also relatively loose. According to Figure 10b–e, when the ATMP and TA were added, a lot of needle-rod-shaped 517 phases were generated, and there was almost no Mg(OH)_2_, indicating that the formation of Mg(OH)_2_ was hindered by ATMP and TA, thus accelerating the formation of 517 phase, which was in accordance with the results of the XRD. With the increased dosage of ATMP, from ATMP-2 (Figure 10b) to ATPM-6 (Figure 10d), it was found that the 517 phase was transferred into a short rod-shaped structure, and it seemed to be densified. Furthermore, with the same mole dosage of H^+^, compared to TA-6 (Figure 10e), the more regular crystallization of the 517 phase was observed in ATMP-2 (Figure 10b). With the same mole dosage, compared to TA-6 (Figure 10e), a larger amount of short rod-shaped 517 phase was found in ATMP-6 (Figure 10d).

### 3.6. Effect of ATMP and TA on pH Value of MOS

**Figure 11:**
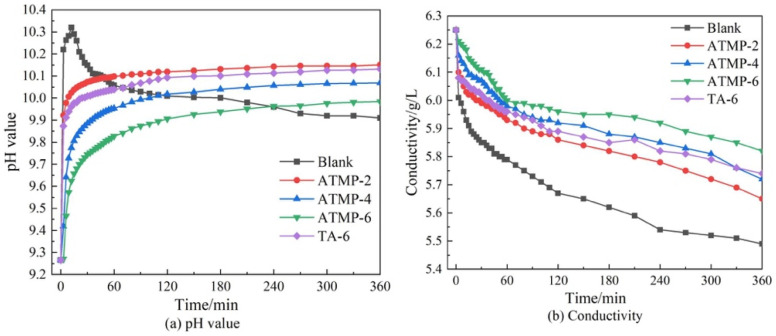
Effect of ATMP and TA on pH value and conductivity of MOS.

The pH value and conductivity of MOS suspension in the presence of ATMP and TA were measured, and the results are shown in Figure 11a. According to the figure, during the hydration process of the blank group, the pH of MOS initially rose rapidly to a peak value (pH = 10.33) and then gradually decreased. This behavior was attributed to the rapid hydrolysis of MgO in the early stage of hydration, and this would release an amount of OH^−^ with a contribution to the pH value. Once the OH^−^ concentration reached a certain level, a reaction between OH^−^ and Mg^2+^ to form Mg(OH)_2_ happened and the precipitates appeared, and this would consume OH^−^ and lead to a subsequent decrease in pH; the conductivity of the suspension was also reduced significantly, as shown in Figure 11b. Furthermore, with the presence of ATMP and TA, the tendency of the pH value curve was notably changed, and no peak was observed. At the very beginning, the pH value was increased because of the OH^−^ resulting from the dissolution of MgO, while OH^−^ could also be consumed by H^+^ offered by ATMP and TA. Therefore, the pH value of the suspension slowly increased after a few minutes. By contrast, comparing ATMP-2 to TA-6, these two had the same mole dosage of H^+^, but ATMP-2 displayed a lower pH value than that of ATMP, and this is due to the difference in anions which could chelate with Mg^2+^, influencing the hydration of MgO. With the same mole dosage of anions, comparing ATMP-6 to TA-6, a much lower pH value was observed in ATMP-6; one reason was that ATMP-6 introduced a larger amount of H^+^ to react with OH- resulting from the hydration of MgO. Another reason is that ATMP-6 showed a greater chelating ability with Mg^2+^ to delay the hydration of MgO. As shown in Figure 11b, ATMP and TA reduced the conductivity compared with the blank group; one reason was due to the reaction between H^+^ and OH^−^, and the other reason was attributed to the chelation between Mg^2+^ and the anions of ATMP and TA.

**Figure 12:**
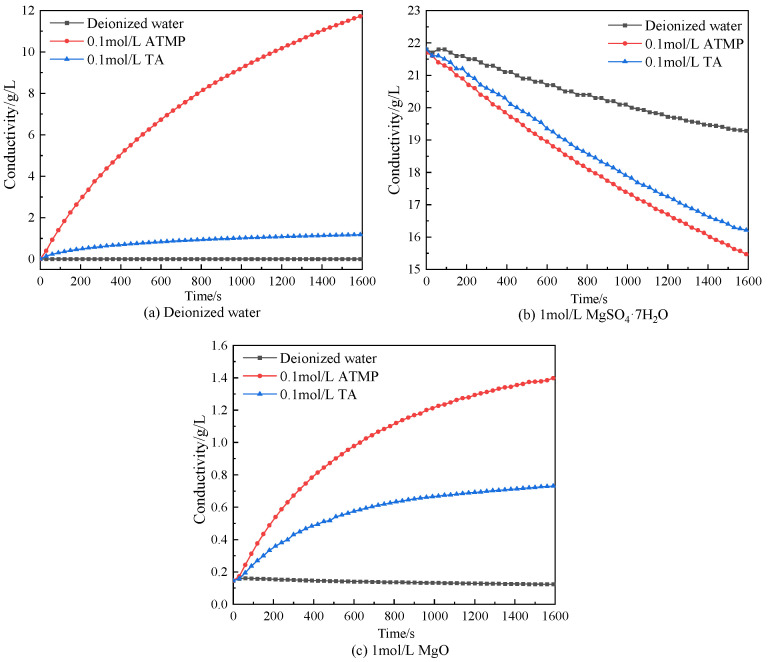
Conductivity of ATMP and TA solutions with MgO and MgSO_4_.

To investigate the chelation of Mg^2+^, the conductivity of the ATMP and TA solutions was measured; the chelation between Mg^2+^ and anions from ATMP and TA was discussed, and the results are presented in Figure 12. Figure 12a shows the conductivity curve of deionized water with continuously added deionized water, 0.1 mol/L ATMP, and 0.1 mol/L TA. It was found that 0.1 mol/L ATMP and 0.1 mol/L TA obviously increased the conductivity of deionized water because of the ionized ATMP and TA with H+ and anions, as shown in Equations (2) and (3). Figure 12b shows the conductivity curve of 1 mol/L MgSO_4_ with continuously added deionized water, 0.1 mol/L ATMP, and 0.1 mol/L TA. It was seen that the continuous decrease in conductivity was observed in the deionized water group, and this was because the MgSO_4_ solution was diluted by adding deionized water. It was also found that the conductivity of the 0.1 mol/L ATMP and 0.1 mol/L TA groups was lower than that of deionized water. If there is no reaction between Mg^2+^ and ATMP or TA, the 0.1 mol/L ATMP and 0.1 mol/L TA groups should be higher than that of deionized water because of the ionization of ATMP and TA contributing to the release of H^+^ and anions. The opposite results confirmed the reaction between Mg^2+^ and ATMP (or TA). By contrast, the conductivity of the 0.1 mol/L ATMP group was lower than that of the 0.1 mol/L TA, and this indicated the stronger chelation ability of TAMP than that of TA with the same mole dosage. Figure 12c shows the conductivity curve of the 1 mol/L MgO suspension with continuously added deionized water, 0.1 mol/L ATMP, and 0.1 mol/L TA. It was seen that a slight decrease in conductivity was observed in the deionized water group because of dilution with deionized water, while for both 0.1 mol/L ATMP and 0.1 mol/L TA, conductivity was increased because of the ionization of ATMP and TA. Based on the discussion above, chelation of ATMP and TA with Mg^2+^ could be confirmed, and by contrast, the chelating ability of ATMP was much higher than that of TA with the same mole dosage.

(1) $$MgO+{2H}^{+}\to {Mg}^{2+}+H_{2}O$$

(2) $$ATMP\overset{H_{2}O}{↔}\;\;{ATMP}^{n-}+n{\;H}^{+}$$

(3) $$TA\overset{H_{2}O}{↔}\;\;{TA}^{m-}+m{\;H}^{+}$$

(4) $${2ATMP}^{n-}+n\;{Mg}^{2+}\to \;\;n\;{Mg}^{2+}\cdot {2ATMP}^{n-}$$

(5) $${2AT}^{m-}+m\;{Mg}^{2+}\to \;\;m\;{Mg}^{2+}\cdot {2TA}^{m-}$$

### 3.7. Mechanism

**Figure 13:**
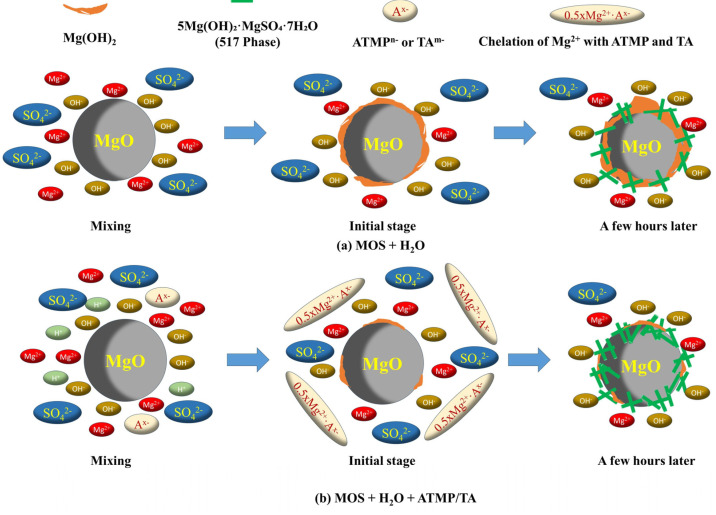
Hydration process of MOS with and without ATMP and TA.

Based on the discussion above, it was found that both ATMP and TA could improve the fluidity, delay the setting time, increase the mechanical strength, and enhance water resistance. The reason being that ATMP and TA hindered the hydration in the initial few hours while they accelerated the hydration later, which was proved by the results of the hydrate analysis, hydration heat, and the pH value. The mechanism behind the initial hindrance and later acceleration was closely related to two aspects: (1) H^+^ from ATMP and TA to hinder the generation of Mg(OH)_2_; (2) chelation with Mg^2+^ in the liquid phase resulting in a supersaturated solution with a higher saturation, which also hindered the generation of Mg(OH)_2_ and then accelerated the generation of the 517 phase. In the first few minutes, because of the dissolution of MgO to release OH^−^ and Mg^2+^, the pH value increased immediately, followed by the generation of Mg(OH)_2_. It is worth noting that most of the Mg(OH)_2_ appeared on the surface of MgO, which would hinder the release of OH^−^ from MgO particles [32,33]. In this process, due to the higher content of H^+^ ions in ATMP, its effects on delaying the setting time and improving fluidity are more pronounced than TA at the same dosage. With time going on, the 517 phase was produced slowly, as shown in Figure 13a. In the presence of ATMP and TA, the generation of Mg(OH)_2_ could be hindered by H^+^ from ATMP or TA, and this would accelerate the dissolution of Mg^2+^, as shown in Equation (1). Because of the strong chelating ability of ATMP and TA, as shown in Equations (4) and (5), a large amount of Mg^2+^ remained in the liquid phase rather than precipitating, which led to the Mg(OH)_2_ phase being produced. In this case, a supersaturated solution with a higher saturation was formed. When the concentration of H^+^ declined to a certain level, the formation of the 517 phase happened, as presented in Figure 13b. By contrast, with the same mole dosage of H^+^, ATMP-2 displayed a higher compressive strength than TA-6. This was attributed to the stronger chelating ability of ATMP than that of TA, which more effectively inhibited the formation of Mg(OH)_2_ and promoted the formation of the 517 phase. Specifically, ATMP contains multiple phosphonic acid groups (-H_2_PO_3_) and an amino group (-NH_2_) in its molecular structure, providing more active sites for coordination with Mg^2+^, whereas TA only has two carboxyl groups (-COOH) as potential chelating sites. This greater number of chelating sites in ATMP allows for more effective sequestration of Mg^2+^ in the liquid phase, thereby more strongly suppressing Mg(OH)_2_ formation and facilitating the preferential growth of the 517 phase, which ultimately contributes to the higher compressive strength observed. These results further indicated that the chelating ability of the anion played a key role in both the retarding effect and performance enhancement.

## 4. Conclusions

The effect of ATMP and TA on the fluidity, compressive strength, hydration process, and hydrates of MOS was comparatively investigated, and the conclusion was drawn as follows:

(1) Both ATMP and TA could improve the fluidity, delay the setting process, increase the strength, and enhance water resistance. The reason being that ATMP and TA hindered the hydration in the initial few hours while they accelerated the hydration later, which was proved by the results of analyzing the hydrates, hydration heat, and the pH value. The mechanism behind the initial hindrance and later acceleration was closely related to two aspects: H^+^ from ATMP and TA to hinder the generation of Mg(OH)_2_; chelation with Mg^2+^ in the liquid phase resulting in a supersaturated solution with a higher saturation, which also hindered the generation of Mg(OH)_2_ and then accelerated the generation of the 517 phase.
(2) By contrast, with the same mole dosage of H^+^, MOS with ATMP-2 displayed a higher compressive strength than TA-6, and with the same mole dosage, ATMP demonstrated greater efficacy in improving fluidity, delaying the setting process, enhancing the compressive strength, and improving water resistance. This was because the chelating ability of ATMP was stronger than that of TA in hindering the formation of Mg(OH)_2_ and then inducing the generation of the 517 phase, and these results also indicated that the chelating ability of the anions played a key role in both the retarding effect and performance enhancement.

## Figures and Tables

**Table 1:** Mix ratio of MOS cement **.

Sample	Mole Ratio				
	MgO	MgSO_4_·7H_2_O	H_2_O	ATMP	TA
Blank	4	1	16	0	0
ATMP-2	4	1	16	0.002	0
ATMP-4	4	1	16	0.004	0
ATMP-6	4	1	16	0.006	0
TA-6	4	1	16	0	0.006

** It was noted that one mole of ATMP contains six moles of H^+^, while TA contains two moles. This indicates that the ATMP-2 group contributes the same mole amount of H^+^ as the TA-6 group.

**Table 2:** Hydration heat of MOS within 48 h.

Scheme		Hydration Heat (J/g)			
	4 h	10.8 h	24 h	30 h	48 h
Blank	68.04	140.07	157.50	159.43	148.66
ATMP-2	9.77	98.79	173.68	178.54	186.88
ATMP-4	6.54	59.14	178.73	188.06	192.31
ATMP-6	4.92	30.81	172.25	189.68	201.26
TA-6	33.24	140.07	163.97	166.71	165.65

## Data Availability

Dataset available on request from the authors. The raw data supporting the conclusions of this article will be made available by the authors on request.
